# *Operando* acoustic emission monitoring of degradation processes in lithium-ion batteries with a high-entropy oxide anode

**DOI:** 10.1038/s41598-021-02685-2

**Published:** 2021-12-03

**Authors:** Simon Schweidler, Sören Lukas Dreyer, Ben Breitung, Torsten Brezesinski

**Affiliations:** Institute of Nanotechnology, Karlsruhe Institute of Technology (KIT), Hermann-von-Helmholtz-Platz 1, 76344 Eggenstein-Leopoldshafen, Germany

**Keywords:** Batteries, Batteries, Energy

## Abstract

In recent years, high-entropy oxides are receiving increasing attention for electrochemical energy-storage applications. Among them, the rocksalt (Co_0.2_Cu_0.2_Mg_0.2_Ni_0.2_Zn_0.2_)O (HEO) has been shown to be a promising high-capacity anode material. Because high-entropy oxides constitute a new class of electrode materials, systematic understanding of their behavior during ion insertion and extraction is yet to be established. Here, we probe the conversion-type HEO material in lithium half-cells by acoustic emission (AE) monitoring. Especially the clustering of AE signals allows for correlations of acoustic events with various processes. The initial cycle was found to be the most acoustically active because of solid-electrolyte interphase formation and chemo-mechanical degradation. In the subsequent cycles, AE was mainly detected during delithiation, a finding we attribute to the progressive crack formation and propagation. Overall, the data confirm that the AE technology as a non-destructive *operando* technique holds promise for gaining insight into the degradation processes occurring in battery cells during cycling.

## Introduction

Lately, a new class of multicomponent ceramics, high-entropy materials (HEMs), is receiving attention for rechargeable lithium-ion battery (LIB) applications^1–4^. In general, HEMs rely on the high-entropy concept, that is, the introduction of a large number of elements in a single-phase structure to increase the configurational entropy^5^. This typically leads to strong interactions between the incorporated elements, so-called cocktail effects, and in some cases, to an entropy stabilization. The fact that HEMs can be tailored by compositional design, among others, makes them so exciting and versatile. A series of different HEMs have been synthesized successfully, including oxides, borides, carbides, nitrides, sulfides and silicides^5–9^. Both composition and structure are known to strongly determine their properties and therefore the field of application^3,4,6,10,11^.

A promising high-entropy oxide anode material is the rocksalt (Co_0.2_Cu_0.2_Mg_0.2_Ni_0.2_Zn_0.2_)O, which is referred to as HEO hereafter^1–3,12^. The HEO electrode is capable of delivering stable specific capacities of *q* > 350 mAh/g_HEO_ in LIB cells^1,13^. Previous investigations using X-ray diffraction (XRD), selected-area electron diffraction (SAED) and transmission electron microscopy (TEM) have shown that the lithiation/delithiation processes rely on a partial conversion mechanism. This means that only some of the cations are reduced to the elemental state upon lithiation (as expected for a conversion reaction) while the others retain the rocksalt structure to some degree. This combined mechanism appears to be the main reason for the good cycling stability of the material. In the initial lithiation cycle, the XRD reflections stemming from the HEO lattice vanished^1^, which is characteristic of conversion materials and can be explained by the formation of either amorphous or nanocrystalline domains^14,15^. However, the crystal structure was still detectable by ex situ SAED. Recent X-ray absorption spectroscopy (XAS) studies have shown that the initial lithiation/delithiation processes are incomplete and irreversible^16^, leading to the formation of a mixture of metals and metal oxides, and that alloying and dealloying reactions with Li are involved in the charge-storage mechanism in the subsequent cycles (typical of ZnO and MgO anodes). Overall, this somewhat limits the possibility of *operando* investigations beyond the first cycle and, in particular, hinders further understanding of the reaction and degradation mechanisms under realistic conditions.

To gain more insight into the behavior of the HEO anode during electrochemical cycling, the acoustic emission (AE) technology is employed here. AE is particularly suited, as it is a non-destructive method applicable in real-time. It has already been used in the investigation of various battery materials such as graphite, Si, LiCoO_2_ (LCO), LiNiO_2_ (LNO) or NiSb_2_^17–24^. By classifying the acoustic signals, conclusions could be drawn about the insertion mechanism, the SEI formation and the mechanical degradation^20,23,25–27^. Herein, we report an *operando* AE study of HEO/Li cells. The data analysis is partly based on discussions from related literature reports, in which the electrochemical, structural/morphological and gassing behaviors were thoroughly investigated^1,2,13^. Overall, the purpose of this work is to advance understanding of the degradation processes occurring in HEO-based LIB cells, which may help to improve the overall charge-storage properties of electroactive HEMs in the long run. In addition, this work aims at improving knowledge of AE monitoring in the field of *operando* characterization of battery materials.

## Materials/methods

### Materials and testing

The (Co_0.2_Cu_0.2_Mg_0.2_Ni_0.2_Zn_0.2_)O (HEO) anode material was synthesized by nebulized spray pyrolysis, as described elsewhere^28^. The electrodes were prepared by water-based slurry coating onto Cu foil. They consisted of 63 wt% HEO active material, 22 wt% Super C65 carbon black additive (Timcal) and 15 wt% Selvol 425 poly(vinyl alcohol) binder (Sekisui) and had an average loading of 2.5 mg_HEO_/cm^2^. Electrochemical testing and *operando* AE measurements were done on CR2032 coin cells. The cells were assembled in an Ar-filled glovebox by stacking the HEO working electrode (12 mm diameter), GF/D glass microfiber separator (17 mm diameter; GE Healthcare Life Science, Whatman) soaked with 100 µL LP57 electrolyte (1 M LiPF_6_ in a 3:7 mixture by weight of ethylene carbonate and ethyl methyl carbonate) and Li metal counter-electrode (15 mm diameter; Albemarle Germany GmbH). They were cycled at a rate of C/10 (1C = 1000 mA/g_HEO_) and 25 °C between 0.01 and 2.5 V versus Li^+^/Li using a BAT-SMALL potentiostat (Astrol Electronic AG).

### Acoustic emission

The AE setup consisted of a differential wideband sensor (125–1000 kHz; MISTRAS Group, Inc.), an in-line preamplifier and a data acquisition system (USB AE Node, MISTRAS Group, Inc.). The signals were filtered (100–1000 kHz), amplified (40 dB gain) and processed by the acquisition system. To prevent acoustic signals within the background noise, a threshold value of 27 dB was set after performing blind tests. Signals of less than two counts or < 100 kHz were eliminated. The sensor coupling was probed using the pencil-lead break test^29^. For the calculation of hit rate and more details on the AE analysis, the reader is referred to the literature^17,20,21,27,30,31^.

### Scanning electron microscopy

SEM imaging was done using a LEO-1530 electron microscope (Carl Zeiss AG).

## Results and discussion

**Figure 1:**
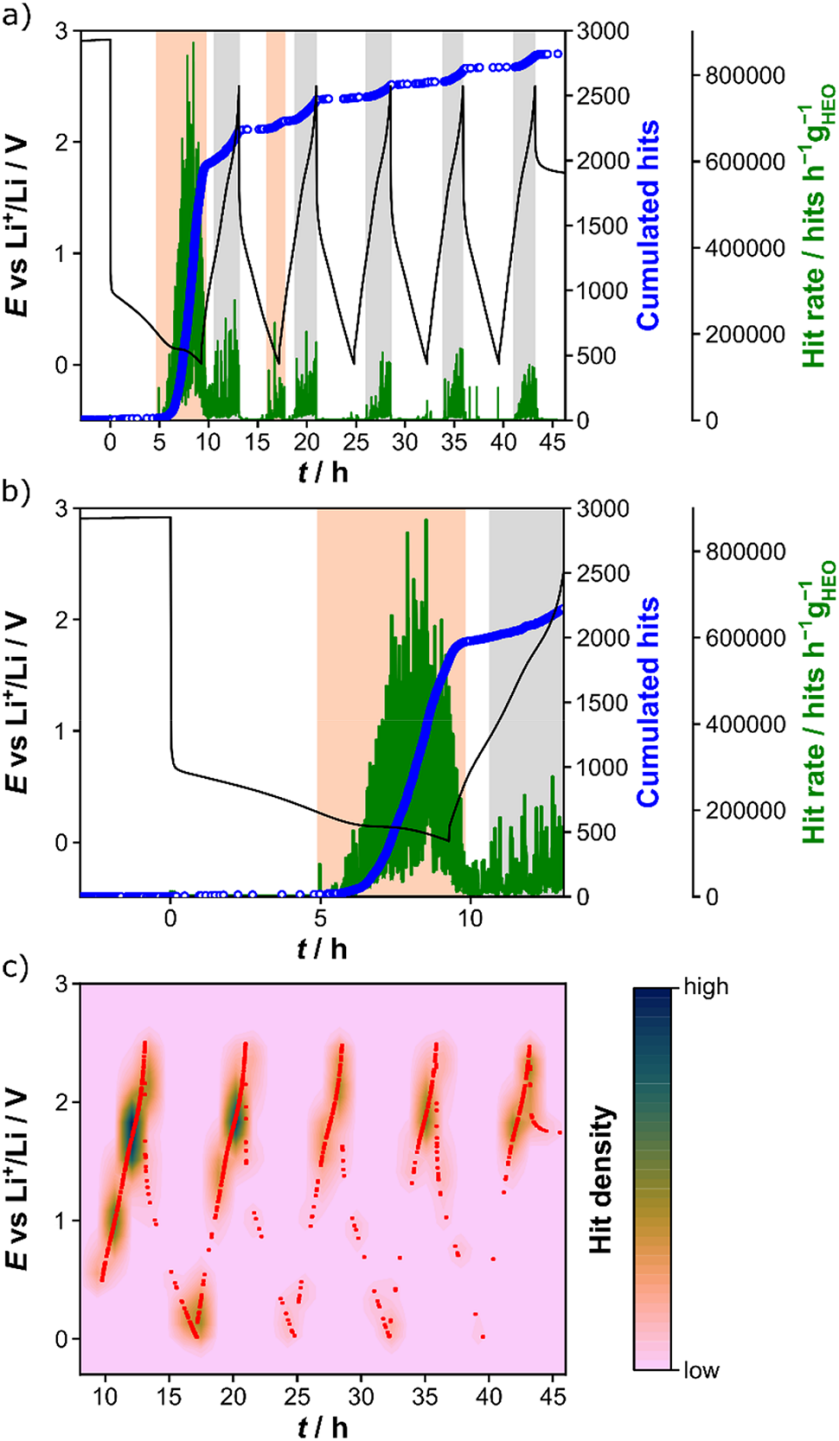
(**a**) Voltage profile for the first five cycles (black) and corresponding cumulated hits (blue) and hit rate (green). (**b**) Enlarged view of the first cycle. Orange and gray shaded areas indicate major acoustic activity during lithiation and delithiation, respectively. (**c**) Contour plot of acoustic activity (hit density) as a function of time and voltage (starting from the initial delithiation cycle at 0.5 V vs Li^+^/Li). Hits are denoted by red dots.

Figure 1a shows the voltage profile of the HEO half-cell for the first five cycles together with the cumulated hits and calculated hit rate. The strongest acoustic activity was observed in the initial cycle, with specific capacities of 926 mAh/g_HEO_ (lithiation) and 382 mAh/g_HEO_ (delithiation), which will be discussed in more detail below. In general, the charge/discharge curves and the values for the specific capacity resemble previously reported data^13^. The lithiation and delithiation capacities for all five cycles are provided in Table S1 (Supporting Information).

Interestingly, no AE was detected in the early stages of lithiation. Only when *E* approached ~ 0.3 V vs Li^+^/Li, there was a sudden constant increase in acoustic activity (see cumulative hits and hit rate in Fig. 1a,b). Its appearance in the voltage range between 0.3 and 0.01 V agrees well with findings from previous studies on the same material^1^. For example, using *operando* XRD, Sarkar et al. found that the initial rocksalt structure vanishes in the initial lithiation cycle at ~ 0.3 V^1^. Prior to this, the XRD reflections remained virtually unaffected, which in turn means that there are no major changes in lattice parameters or unit-cell volume, and thus presumably no mechanical degradation occurs. In general, the disappearance of reflections in the XRD pattern is to be expected for a conversion material, since smaller crystallites may form that are below the XRD detection limit^14,15^. Accordingly, it can be assumed that the increase in acoustic activity at *E* ≤ 0.3 V is due to the conversion reaction itself and the associated deformation of the crystal lattice^32^. In particular, the strong volumetric changes with Li uptake can lead to mechanical deformation, including particle fracture and pulverization, which strongly impacts on the AE^20,21,27,31^. Note that major contributions of the Li anode to the acoustic activity can be neglected^17,31,33^. Furthermore, investigations into the gassing behavior of HEO half-cells by Breitung et al. revealed that gas evolution (H_2_ and C_2_H_4_) due to reductive electrolyte decomposition starts at *E* ≈ 0.37 V in the initial cycle, with a maximum at the lower cutoff voltage of 0.01 V^13^. However, the detection of both the formation and bursting of gas bubbles can be largely ruled out based on previous results^31,34^. Even if the outgassing can be excluded as a source of significant acoustic activity, the evolution of C_2_H_4_ is a clear indication of the formation of a solid-electrolyte interphase (SEI) on the anode^13,35,36^. Both SEI formation and cracking are acoustically intense and detectable processes, as demonstrated by various AE studies^17,19,20,31,33^. The assignment to SEI formation is further supported by the fact that the appearance of acoustic activity in the second lithiation cycle is also consistent with the evolution of C_2_H_4_ at *E* ≤ 0.2 V^13^. For the subsequent cycles, minor or no AE was recorded near the cutoff voltage. The strong increase in acoustic activity in the initial lithiation cycle can therefore be attributed to both side reactions with the carbonate-based electrolyte and mechanical degradation induced by the charge-storage reactions^17,19,20,31,33^.

**Figure 2:**
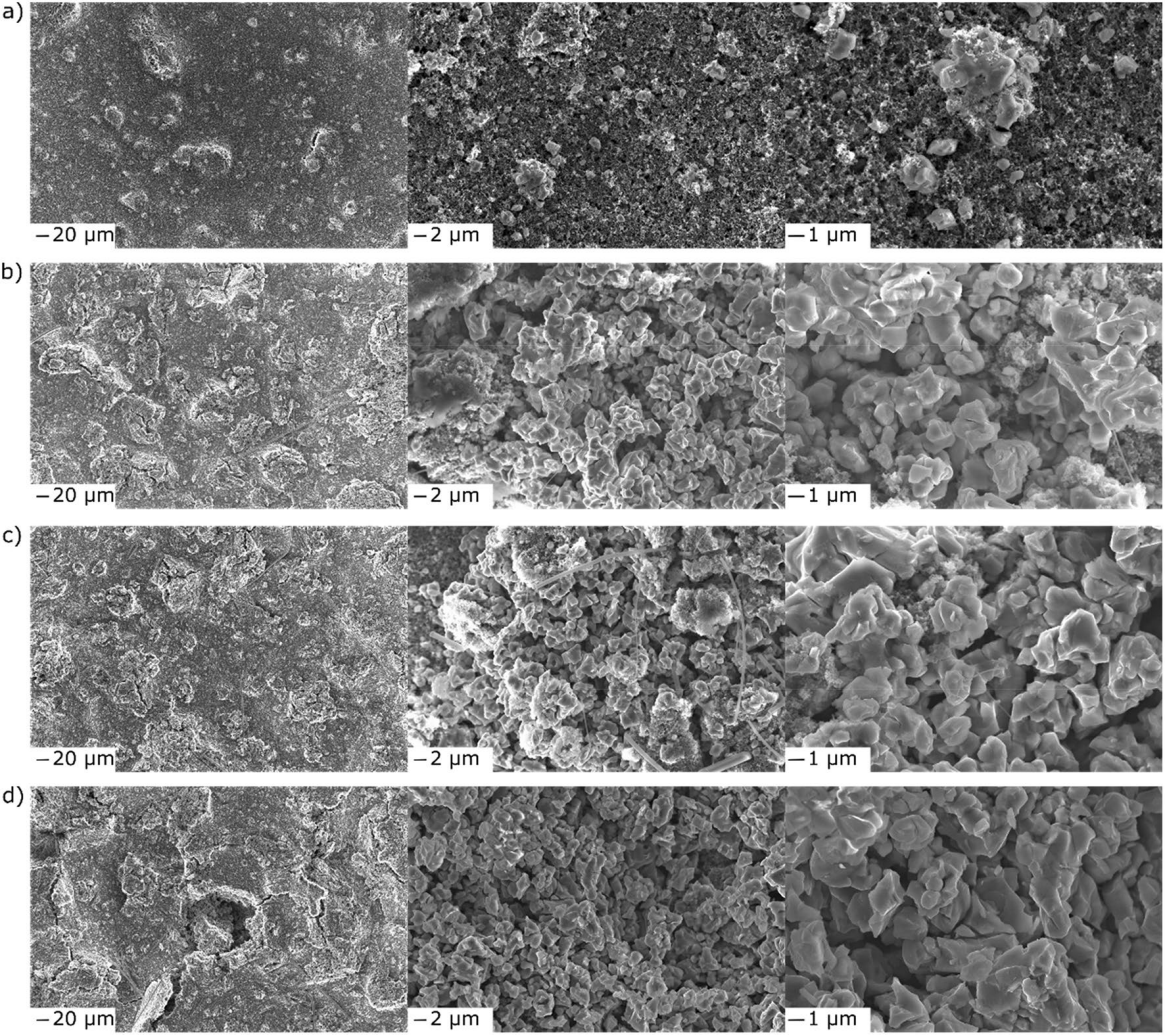
Top-view SEM images at different magnifications of the HEO anode (**a**) before cycling and (**b**) at 0.15 V, (**c**) 0.12 V and (**d**) 0.01 V vs Li^+^/Li in the initial lithiation cycle.

Figure 2a–d shows SEM images at different magnifications collected before, during and at the end of the first lithiation cycle. As can be seen from Fig. 2a, the pristine electrode was porous, as expected, and free of microcracks. The higher-magnification images indicate the presence of scattered agglomerates on the top surface. However, the vast majority of the HEO material was incorporated well in the anode, aggravating more detailed characterization by means of SEM. A kind of localized, crater-like cracking was observed upon electrochemical cycling (Fig. 2b), which increased with increasing degree of lithiation (Fig. 2c). In addition, pulverization of the HEO agglomerates was evident, leading to loosened bulk material with much smaller agglomerates and even individual primary particles. Some electrode cracking was to be expected, since conversion and alloying reactions are usually accompanied by large volumetric changes^32^. The profound effect that such fracturing/cracking has on the acoustic behavior has been shown, e.g., for silicon and metal hydride electrodes^20–22^. The presence of loose material and the resulting contact loss help to explain the drop in specific capacity after the initial cycle. At the cutoff voltage, more cracks and craters appeared (Fig. 2d), suggesting continuous active material loss and (surface/bulk) electrode restructuring. Unfortunately, the SEI formation could not be visualized; the SEI layer on the HEO particles was apparently too thin to be observed with SEM. However, it was already verified by the aforementioned gassing study (i.e., evolution and release of C_2_H_4_)^13^.

Upon delithiation, distinct AE was detected at first before decreasing sharply and then increasing again at ~ 0.9 V (Fig. 1b). One possible explanation for the sudden decay might be some kind of homogenization of the Li concentration within the active material^27^. From 0.9 V to the upper cutoff voltage of 2.5 V, there was constant acoustic activity (buildup of tensile stress). In general, it was less intense, indicating that the mechanical degradation (particle fracture, electrode cracking etc.) and SEI formation are most prominent in the initial lithiation cycle. However, the cracks that form during the course of cycling affect the propagation of acoustic waves through the electrode to some (unknown) extent^20^, which may result in a lower overall number of AE signals.

Upon further cycling, the acoustic behavior (in terms of cumulated hits and hit rate) in the delithiation cycles was similar. Comparable observations have been made for Si anodes^34^, thus indirectly corroborating the alloying/dealloying reaction mechanism proposed by Ghigna et al.^16^ Acoustic activity was found in the voltage ranges of 1.0–2.5 V (2nd and 3rd cycles) and 1.3–2.5 V (4th and 5th cycles). The shift to higher voltages with increasing cycle number is also evident from the contour plot in Fig. 1c. It depicts the hit density as a function of time and voltage starting from the first delithiation cycle at 0.5 V (otherwise the informative value for the subsequent cycles would have been lost, see Figure S1, Supporting Information). The contour plot indicates that the first two delithiation cycles, in particular, exhibit intense acoustic activity in the higher voltage range, with two distinct hit-density peaks at ~ 0.9 and 1.7 V and at ~ 1.2 and ~ 1.8 V. In the following cycles, these peak somewhat merged and shifted, as mentioned earlier.

**Figure 3:**
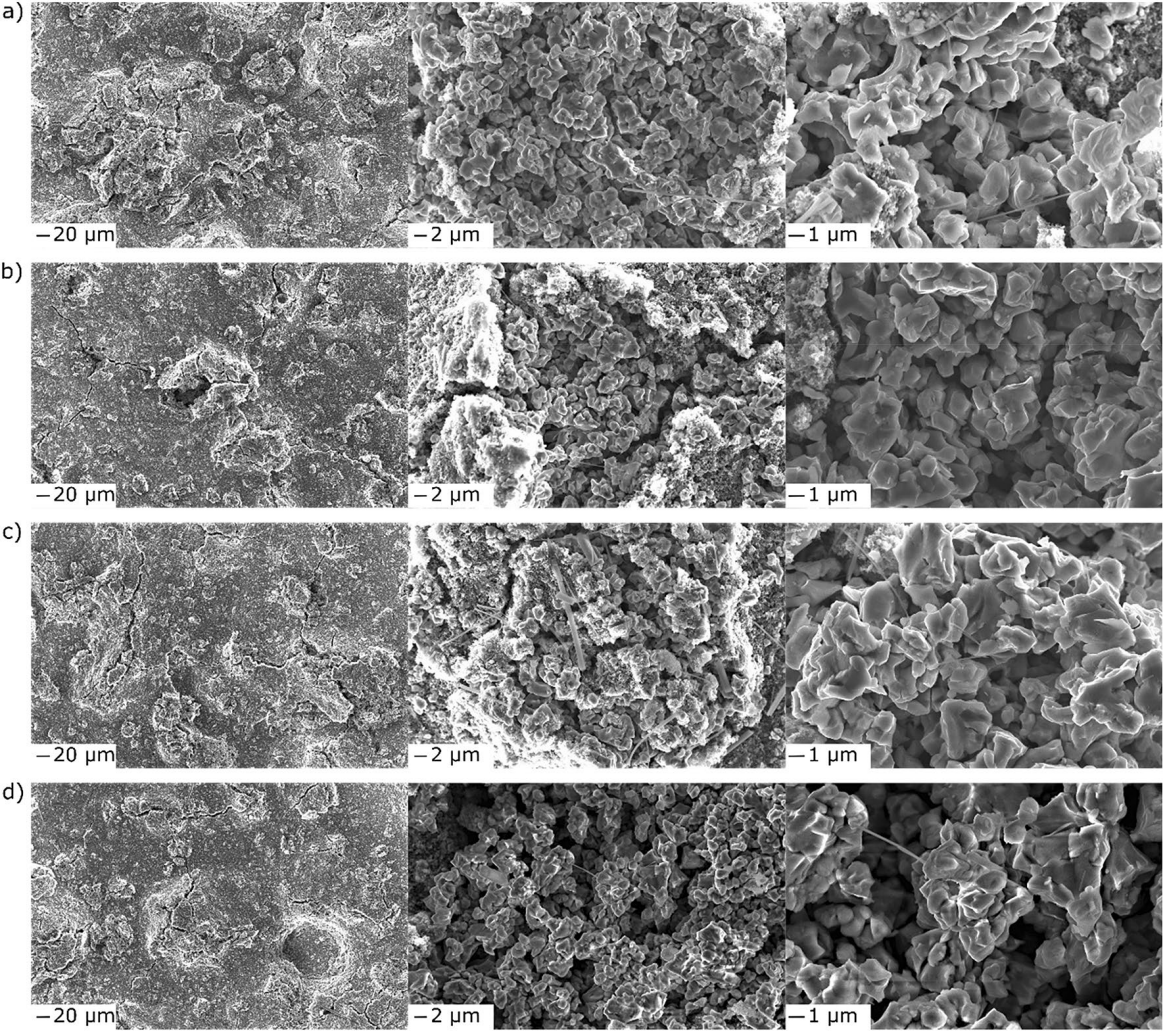
Top-view SEM images at different magnifications of the HEO anode (**a**) at 1.0 V, (**b**) 1.8 V and (**c**) 2.5 V vs Li^+^/Li in the initial delithiation cycle and (**d**) after the first five cycles.

In general, the detection of AE almost exclusively during the delithiation cycles agrees with the mechano-electrochemical modeling study of Barai et al.^37^ They predict a more pronounced mechanical degradation of the active material particles upon delithiation. Specifically, the study suggests that there is an increased buildup of stress on the particle surface relative to that of the core, eventually leading to surface fracture. This provides an explanation for the continuous acoustic activity. However, SEM imaging did not show clear signs of increased particle fracture during the delithiation process (Fig. 3a–c). Thus, it appears that the AE is also due to other effects such as the disintegration of secondary particles and the opening and enlargement of cracks in the electrode (Fig. 3d).

In contrast to the delithiation cycles, virtually no acoustic activity was detected upon lithiation from the second cycle onward. This might be another indication that the HEO material indeed undergoes alloying/dealloying reactions with Li after the initial irreversibilities. In the second cycle, a notable increase was observed at *E* ≤ 0.2 V (Fig. 1a,c), which is consistent with the observation of C_2_H_4_ evolution due to electrolyte decomposition (SEI formation)^13^. Overall, the AE data corroborate the results from the recent gassing study and confirm the formation of a robust SEI on the HEO particles during the initial cycles. Comparable acoustic behavior was also seen in repeated *operando* measurements (Figure S2, Supporting Information), with differences in the absolute values of the cumulated hits and hit rate being likely due to differences in cell capacities (Table S1, Supporting Information).

As mentioned previously, AE events can be caused by different phenomena such as SEI formation, electrode cracking, primary particle fracture or secondary particle fragmentation^17,19–21,26,27,31,33^. To probe the correlation between the AE events and their origin, in the present work, the acoustic signals were classified based on the peak frequency. Note that they can be distinguished by a variety of characteristic parameters, including the duration time, rise time, absolute energy, amplitude and/or peak frequency, with the latter being the most prominent (and distinguishable) parameter^20,27,31^. This becomes particularly evident when comparing the mean values of the characteristic AE parameters (Table S2, Supporting Information).

**Figure 4:**
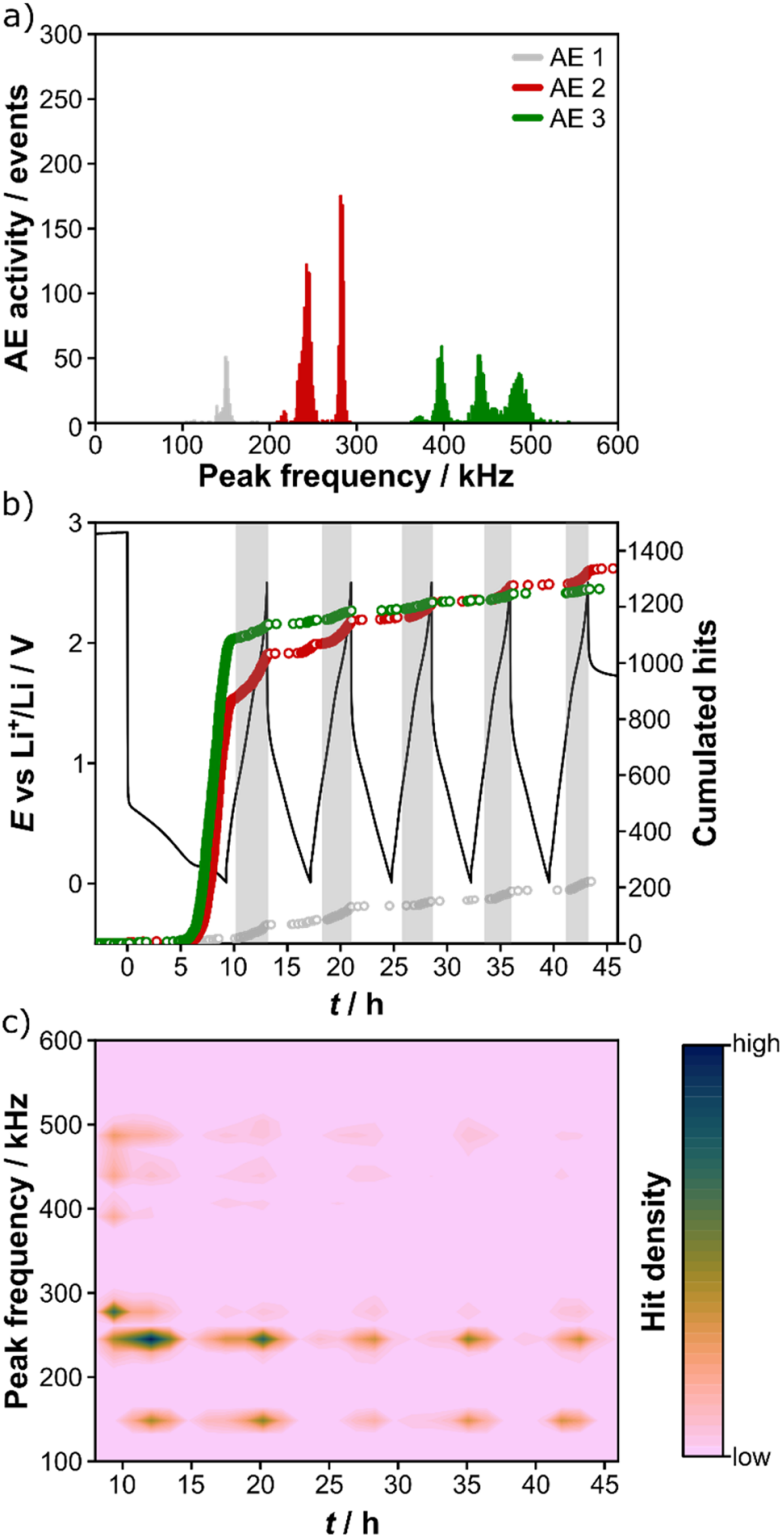
(**a**) Peak-frequency histogram of AE signals detected during the first five cycles. The signals were separated and clustered into different groups based on their peak frequency. (**b**) Voltage profile (black) and the corresponding cumulated hits for the different peak-frequency ranges [same color scheme as in (**a**)]. Major acoustic activity in the delithiation cycles is highlighted for clarity. (**c**) Contour plot of acoustic activity (hit density) as a function of time and peak frequency (starting from the initial delithiation cycle at 0.01 V vs Li^+^/Li).

The acoustic signals were clustered into three groups, AE 1, AE 2 and AE 3 (Figure S3, Supporting Information). Figure 4a shows the peak-frequency histogram for the first five cycles. AE 1 comprises the frequency range of 104–185 kHz, with a peak maximum at ~ 150 kHz. AE 2 and AE 3 cover the frequency ranges of 208–292 kHz and 362–543 kHz, respectively, with peak maxima at ~ 280 and ~ 400 kHz. As can be seen from Fig. 4b, the AE 2 and AE 3 signals accounted for ~ 48 und 44%, respectively, of the total cumulated hits. This is further emphasized by the contour plot in Fig. 4c, showing the hit density as a function of time and peak frequency starting from the initial delithiation cycle. The first lithiation cycle is again excluded for the same reason above (strongest acoustic activity, see Figure S4, Supporting Information).

In the initial lithiation cycle, the AE 3 signals increased sharply when reaching ~ 0.3 V, followed by a similar increase in AE 2 at ~ 0.2 V. In contrast, no significant increase in AE1 signals was observed. The first appearing AE 3 signals, exhibiting the highest mean peak frequency and shortest rise and duration times, are indicative of a rapid formation of cracks on the HEO particle surface (or even particle fracture) and/or in the electrode^20,22,25–27^. However, they may also be the result of disintegration of the initially agglomerated HEO primary particles, as shown in Fig. 2. The AE 2 signals with a lower mean peak frequency, longer rise and duration times but higher absolute energy may have their origin in the growth of larger cracks in the anode. This would be consistent with the SEM results and conclusions drawn in the literature^20,38,39^. It seems probable though that the latter signals also arise from the SEI formation, especially when considering the acoustic activity observed over the first two lithiation cycles. In fact, previous studies have already shown that the peak-frequency range between 200 and 300 kHz is characteristic of both crack formation and SEI formation/gassing^18,20,26,27^. Apart from that, we believe that the minor AE 1 signals are caused by some kind of surface/bulk restructuring during battery operation^20,25–27^.

In the initial delithiation cycle, there was a rise in all three signals, AE 1, AE 2 and AE 3 at 0.85, 0.7 and 0.85 V, respectively. A comparable behavior was seen in the subsequent delithiation cycles, but with mainly AE 1 and AE 2 contributing. However, the increase in total acoustic activity (peak-frequency clustered events) shifted to higher voltages with increasing cycle number (Figure S5 and Table S3, Supporting Information). Nevertheless, AE was always detected at similar (de)lithiation levels (Table S3, Supporting Information). This result thus suggests that the HEO anode is stressed most at Li contents of *x*Li ≤ 2 (ignoring Li consumption due to SEI formation in the initial cycles). The contour plot in Fig. 4c further shows that the increase in acoustic activity in the first and second delithiation cycles, where two distinct hit-density peaks were observed (Fig. 1c), is due to different frequency ranges and therefore different degradation processes (with AE 1 and AE 2 being the most dominant frequency ranges).

From the evolution of the AE 3 signals and their assignment, we conclude that no further significant degradation in terms of active material fracture occurred after the initial cycle, unlike for other battery materials such as Si or LNO^31^. For example, a continuous emission of high-frequency signals (> 400 kHz) was observed during the high-voltage charging of LNO cathodes, which was assigned to detrimental mechanical effects. Accordingly, it seems that, for the HEO material itself, mechanical degradation occurred predominantly in the initial lithiation cycle and electrode cracking plays a major role in the acoustic behavior.

A next step could be to determine the role of the polymer binder and/or current collector in the anode stability and the corresponding AE. Lemarié et al. found clear differences in the acoustic response of sulfur-based electrodes depending on the binder and current collector system used^40^. In addition, it could be beneficial to adjust the voltage window to limit the mechanical degradation, given that the acoustic activity was most pronounced below ~ 0.3 V in the initial cycle and above ~ 1.0 V during delithiation.

## Conclusion

*Operando* AE monitoring has been shown, in the present work, to be a useful tool to gain insight into the degradation behavior of the HEO negative electrode material upon cycling in Li cells (especially because previous studies have not revealed signs of mechanical failure). Using AE technology, the acoustic response during Li insertion and extraction was detected in real-time, and the signals were distinguished by peak frequency and clustered into different groups. The strongest acoustic activity was observed in the initial cycle at low voltages and can be attributed to mechanical degradation effects and SEI formation. In the subsequent cycles, AE was mainly detected during delithiation when the Li content dropped below a “critical” value. Peak-frequency analysis and ex situ SEM imaging indicated that the acoustic activity in this range is due to further electrode cracking and bulk/surface restructuring. Apart from that, the absence of significant (primary) particle fracture and the AE involved confirmed the unique entropy-stabilized Li-storage mechanism. In summary, AE monitoring provided additional information about the degradation of the HEO anode during battery operation. However, when used as a standalone technique, the assignment of signals to specific reactions or mechanisms remains challenging.

## Supplementary Information

Supplementary Information.

## Data Availability

The datasets generated during and/or analyzed during the current study are available from the corresponding author on reasonable request.
