# Supplementary material for: Operando acoustic emission monitoring of degradation processes in lithium-ion batteries with a high-entropy oxide anode
Source: Sci Rep. 2021 Dec 3;11:23381. doi: 10.1038/s41598-021-02685-2 (PMC8642430; doi:10.1038/s41598-021-02685-2)
Supplement: Supplementary file 1 — Supplementary Information. [file 41598_2021_2685_MOESM1_ESM.docx]

Supporting Information

*Operando* Acoustic Emission Monitoring of Degradation Processes in Lithium-Ion Batteries with a High-Entropy Oxide Anode

Simon Schweidler,^1,^* Sören Lukas Dreyer,^1^ Ben Breitung^1^ & Torsten Brezesinski^1^

^1^Institute of Nanotechnology, Karlsruhe Institute of Technology (KIT), Hermann-von-Helmholtz-Platz 1, 76344 Eggenstein-Leopoldshafen, Germany

*Email: [simon.schweidler@kit.edu](mailto:simon.schweidler@kit.edu)


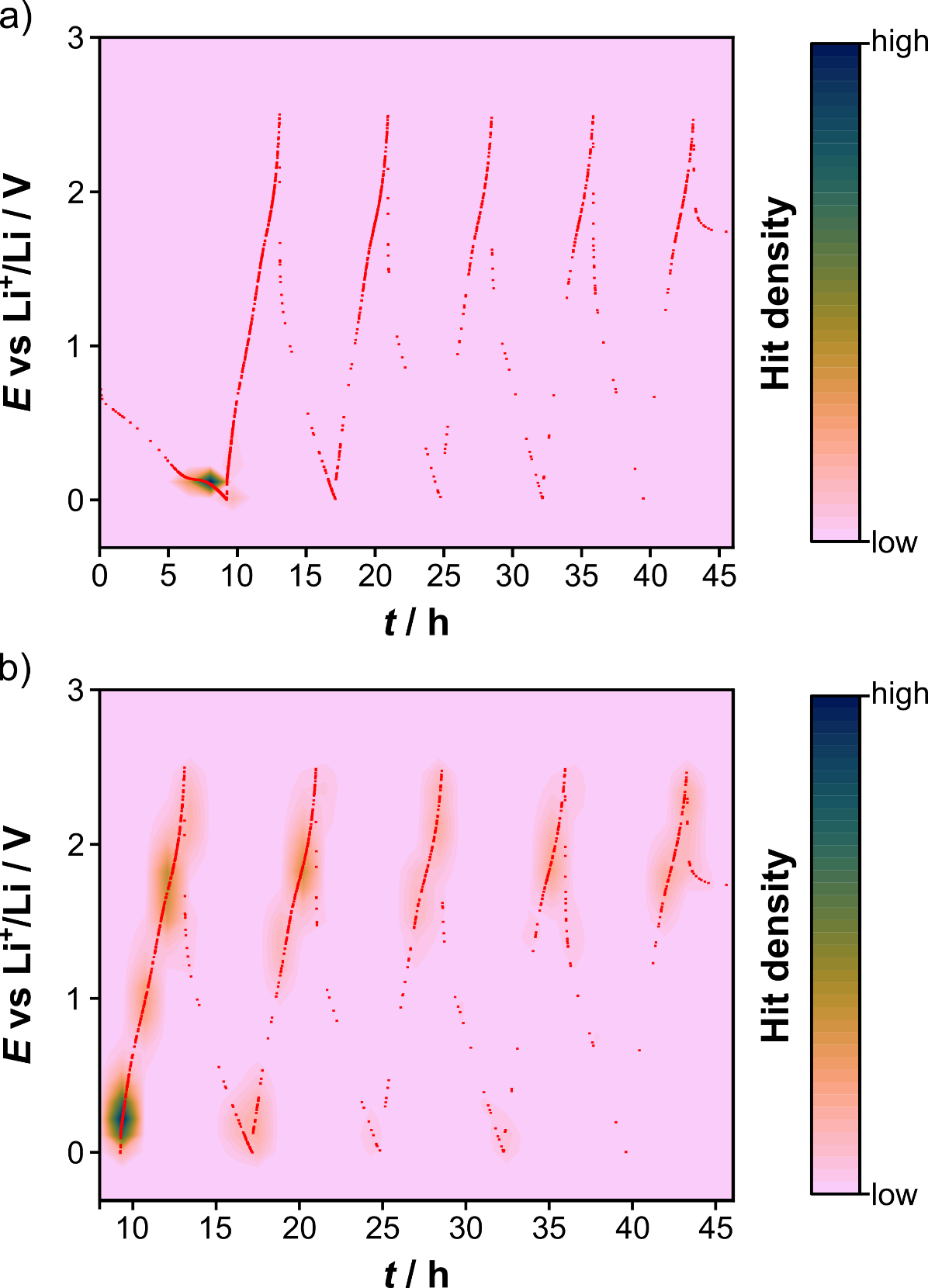


**Figure S1**. Contour plots of acoustic activity (hit density) as a function of time and voltage. (a) Full measurement and (b) starting from the first delithiation cycle at 0.5 V vs Li^+^/Li. Hits are denoted by red dots.


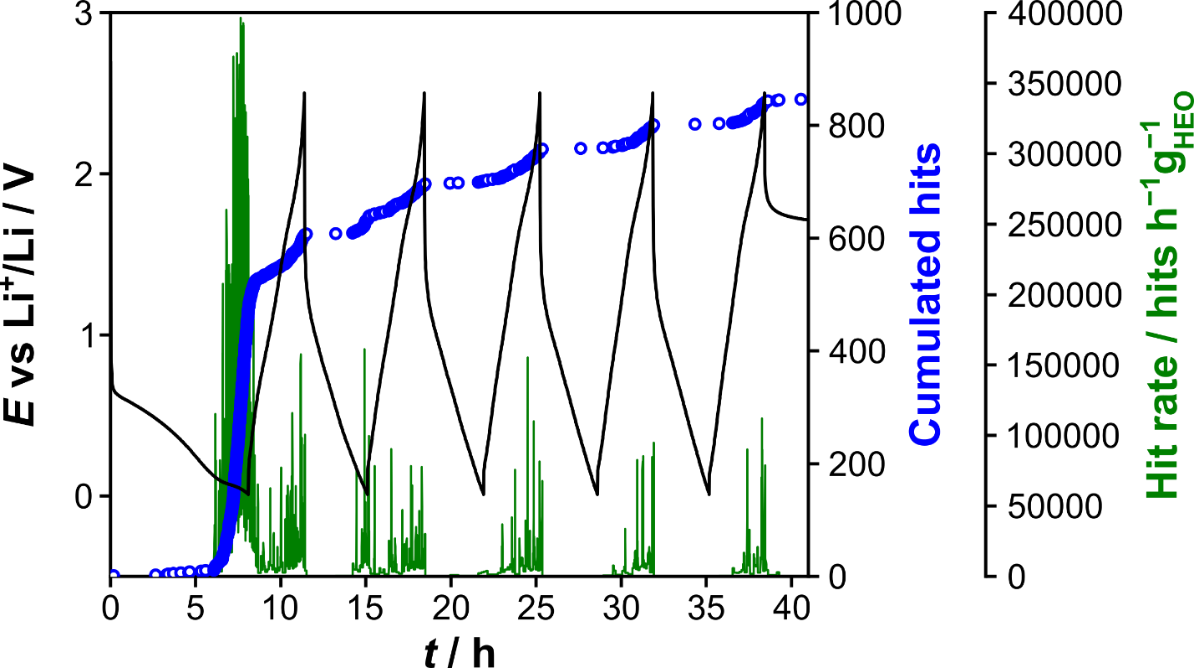


**Figure S2**. Voltage profile for the first five cycles (black) and corresponding cumulated hits (blue) and hit rate (green).


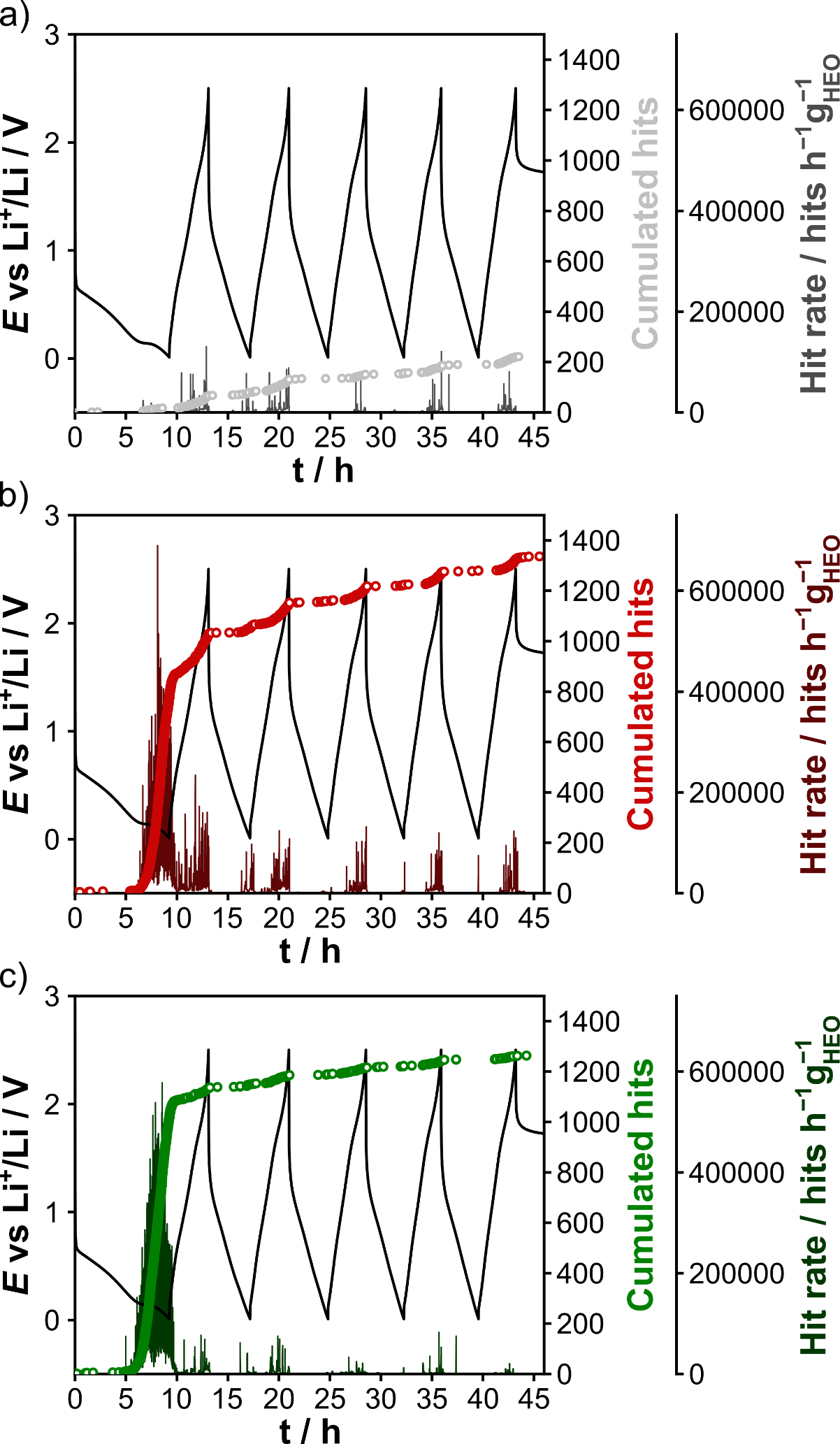


**Figure S3.** Voltage profile for the first five cycles and corresponding acoustic activity (cumulated hits and hit rate) for clustered peak frequency ranges: (**a**) 104-185 kHz, (**b**) 208-292 kHz and (**c**) 362-543 kHz.


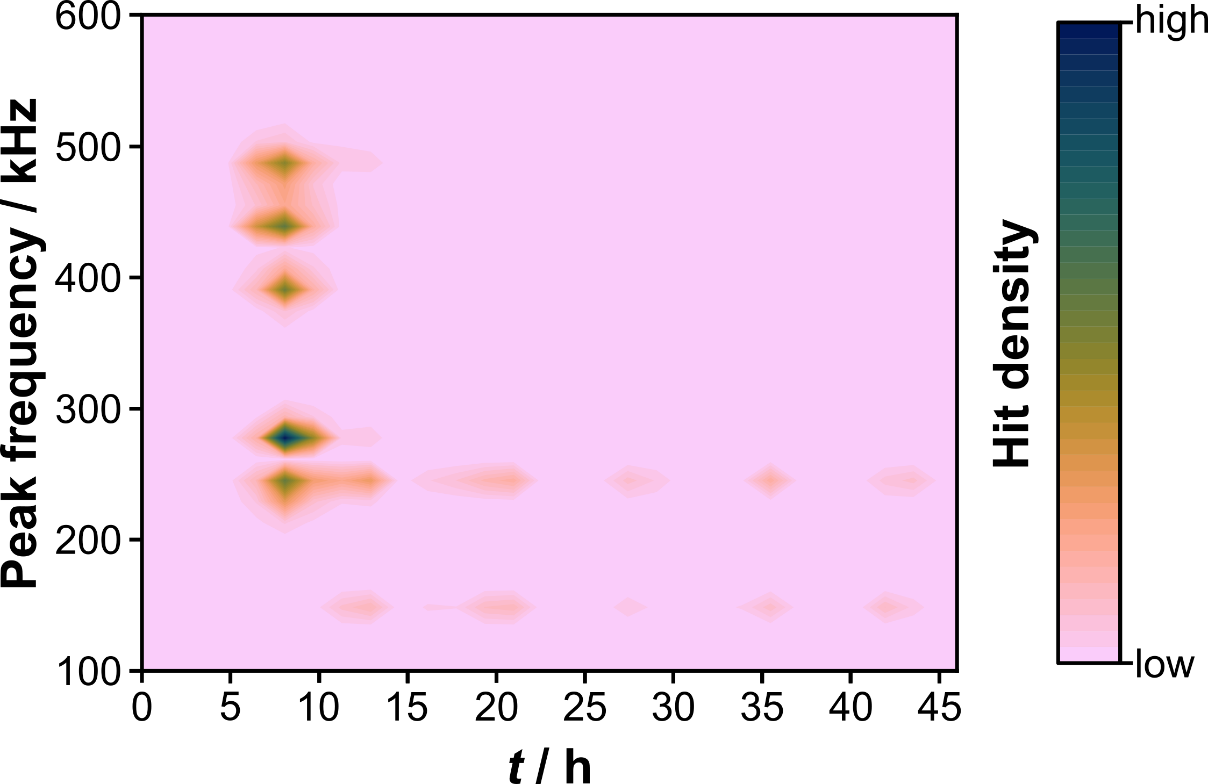


**Figure S4**. Contour plot of acoustic activity (hit density) as a function of time and peak frequency (starting from the first delithiation cycle at 0.01 V vs Li^+^/Li).


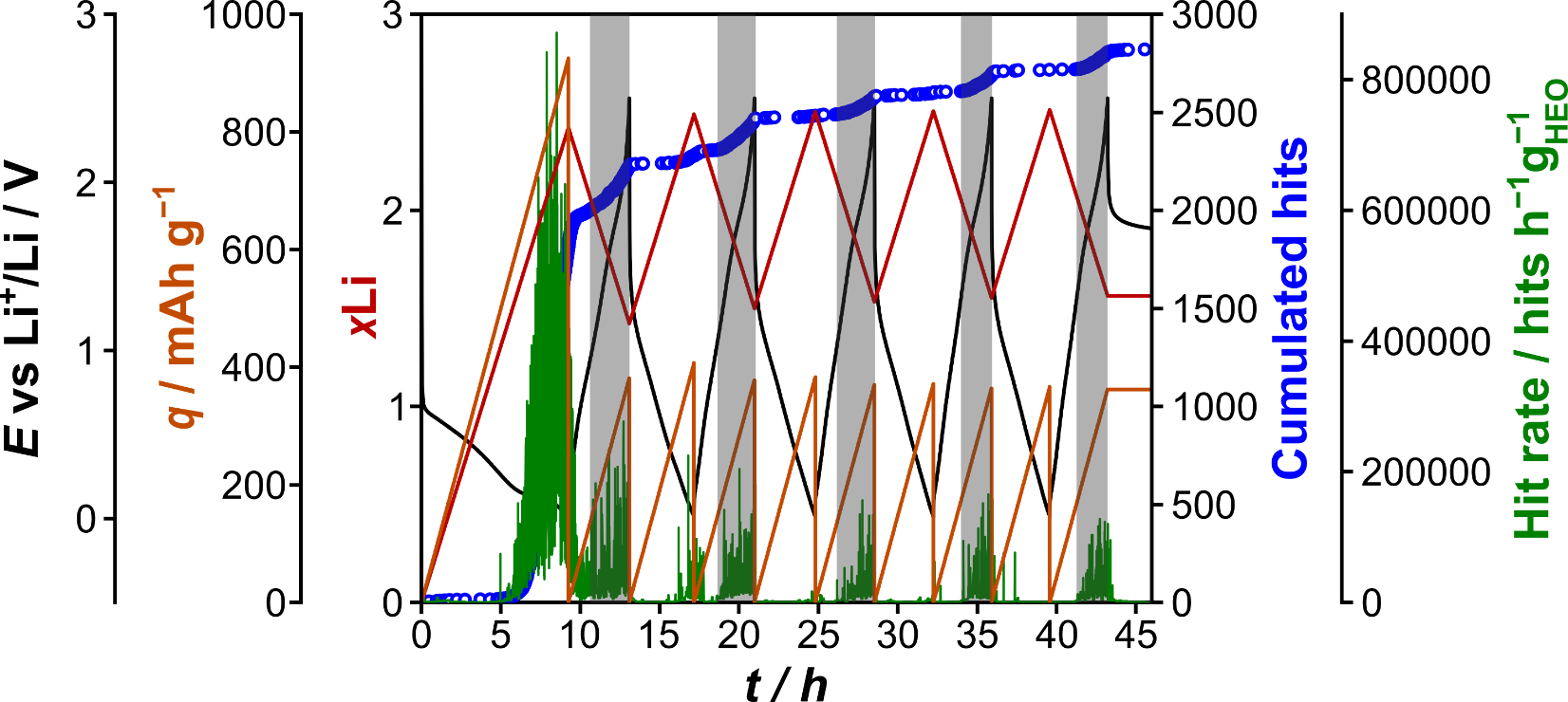


**Figure S5.** Voltage profile (black), specific capacity (orange) and Li content (red) for the first five cycles and the corresponding cumulated hits (blue) and hit rate (green). The acoustic activity in the delithation cycles is highlithed for clarity.

**Table S1.** Comparsion of the specific lithiation and delithiation capacities for the first five cycles of two otherwise identical HEO half-cells. The voltage and acoustic activity profiles in Figure 1 and Figure S2 belong to cell #1 and cell #2, respectively.

|  | |  | **1^st^ cycle** | **2^nd^ cycle** | **3^rd^ cycle** | **4^th^ cycle** | **5^th^ cycle** |
| --- | --- | --- | --- | --- | --- | --- | --- |
| Cell #1 | *q*_lith_ / mAh/g_HEO_ | | 926 | 407 | 383 | 372 | 366 |
|  | *q*_delith_ / mAh/g_HEO_ | | 382 | 378 | 370 | 364 | 362 |
| Cell #2 | *q*_lith_ / mAh/g_HEO_ | | 808 | 370 | 347 | 337 | 331 |
|  | *q*_delith_ / mAh/g_HEO_ | | 329 | 333 | 331 | 325 | 323 |

**Table S2.** Mean values of characteristic parameters for the AE 1, AE 2 and AE 3 signals detected in the first five cycles.

| **Parameter** | **AE 1** | **AE 2** | **AE 3** |
| --- | --- | --- | --- |
| Rise time / µs | 8.32 | 5.22 | 3.14 |
| Duration / µs | 36.90 | 32.09 | 25.45 |
| Amplitude / dB | 34.69 | 36.71 | 37.21 |
| Absolute energy / aJ | 3.14 | 4.78 | 3.90 |
| Peak frequency / kHz | 148.19 | 257.63 | 445.30 |

**Tabel S3.** The starting values for *E*, *q*_delith_ and *x*Li from which significant acoustic activity was detected during the first five delithiation cycles.

| **Cycle no.** | ***E* / V** | ***q*_delith_ / mAh/g_HEO_** | ***x*Li** |
| --- | --- | --- | --- |
| 1 | 0.9 | 143 | 2.04 |
| 2 | 1.1 | 155 | 2.08 |
| 3 | 1.08 | 141 | 2.08 |
| 4 | 1.35 | 149 | 2.03 |
| 5 | 1.36 | 149 | 2.06 |
